# Genetics of cardiovascular disorders: influence of maternal nutrition

**DOI:** 10.1186/1755-8166-7-S1-I5

**Published:** 2014-01-21

**Authors:** Sukhinder K Cheema

**Affiliations:** 1 Department of Biochemistry, Memorial University of Newfoundland, St. John’s, Canada

## 

Cardiovascular disease (CVD) is the number one non-communicable disease of the world. According to the World Health Organization, 30% of global deaths in 2008 were caused by CVD, and it is estimated that by 2030, more than 23 million people will die annually from CVD. Unfortunately, India will be the leading country of the world to have the highest rate of CVD by 2030. Genetics, as well as diet and life style are the predominant factors predisposing the population to an increased risk of CVD. Although genetic makeup is generally considered as the culprit, recent phenomenon of “Developmental origins of health and diseases” or “in-utero programming” places significant importance on maternal diet in predisposing the next generation to metabolic disorders. According to this phenomenon, the diet of the mother during gestation and lactation affects the genetic makeup of the growing foetus, thereby causing perturbations in the metabolic regulations of the offspring, and later onset of diseases. Dietary fats are known to be associated with an increased risk of CVD; however the importance of maternal dietary fats in “in-utero programming” and the onset of CVD in the offspring in later life is not clear. We have shown that a maternal diet high in saturated fatty acids (SFA) increased the levels of low-density lipoprotein (LDL) cholesterol of the offspring by inhibiting hepatic LDL-receptor gene expression. Furthermore, maternal high fat diets caused aortic endothelial dysfunction, which is an additional factor associated with CVD. High fat diets during gestation and lactation also induce fatty liver and myocardium hypertrophy, due to inhibition of beta-oxidation through peroxisome proliferator activated receptors (PPARs). We found that maternal high fat diets altered the gene expression of PPARs, which likely involves epigenetic modifications. On the other hand, we found that maternal diets high in omega (n)-3 PUFA reduced plasma lipid levels of the offspring, thereby reducing the risk of CVD. Our findings have established the importance of maternal dietary fat intake during gestation and lactation to prevent the onset of CVD in the next generation. The Indian population is predisposed to an increased risk of CVD due to their genetic makeup. It is therefore recommended that the Indian population, especially women in their child bearing years, should manage the intake of dietary fat- both the quantity and the quality, to prevent the onset of CVD and other metabolic disorders in the next generation.

